# An impact assessment of insecticides application on the non-targeted mosquito *Aedes albopictus* (Skuse) in Punjab rice fields, Pakistan

**DOI:** 10.7717/peerj.13697

**Published:** 2022-07-11

**Authors:** Hafiz Azhar Ali Khan

**Affiliations:** Department of Entomology, University of the Punjab, Lahore, Punjab, Pakistan

**Keywords:** Ecotoxicology, Insecticide resistance, Fitness cost, Risk assessment

## Abstract

Insecticidal control of insect pests of rice crop may influence the environment and nontarget species in rice fields. *Aedes albopictus*, one of the most common nontarget species present in rice fields, received lethal and sublethal exposures to insecticides used in rice cultivated fields. The present work explores the effects of insecticides in six non-targeted *Ae. albopictus* strains collected from rice fields with a history of insecticidal usage in comparison with a laboratory susceptible reference strain (REF) and a strain (LHR) collected from a rice field with no, or minimal, history of insecticidal usage. Two types of effects, the resistance development and performance of biological traits, were studied by selecting seven commonly used insecticides in rice fields in Punjab, Pakistan. The results revealed that the strains collected from the rice fields with histories of insecticidal usage exhibited significant levels of resistance to flonicamid, chlorantraniliprole, gamma-cyhalothrin, fipronil, monomehypo, triazophos, and carbofuran, when compared with REF and LHR strains. In addition, *Ae. albopictus* strains revealed a significantly weaker performance of biological traits (rate of pupae formation, survival of male and female adults (except females of OKR and MTN strains), and ovipositing females) than those of the REF and LHR strains. However, the fecundity of all field strains was only significantly different with that of the REF strain. In conclusion, the results highlight the problem of the negative effects of insecticidal usage in rice fields on nontarget species present in the same environment, and emphasize the need to adopt pest management activities that are safe for the environment.

## Introduction

Agriculture has been growing at a fast pace in the last few decades, largely due to the modernization of farm machinery and improvement in farm inputs. Of these, insecticides have been one of the major farm inputs that has a significant impact on the increasing yield of different crops (Belchior et al., 2017; Cooper & Dobson, 2007). The use of insecticides that are quick in action and easy to apply has been the preferred choice of control by farming communities, compared to other insect pests control tactics (Khan, 2020b). However, the massive use of insecticides generally results in lethal and sublethal exposures to nontarget species and affects the quality of the environment. (Desneux, Decourtye & Delpuech, 2007; Georghiou, 1990; Khan & Akram, 2017). Hence, the current scenario in developing insect pest management programs usually includes insecticidal measures that can manage insect pests effectively while reducing side effects on nontarget species and the environment (Monteiro et al., 2019).

Besides insect pests, there are a number of nontarget insects present in farming areas that usually receive exposures to insecticide residues during and/or after spray applications through different routes such as drift, runoff, respiration, or occurrence of aquatic insects in standing water in cropping areas, and/or consuming insecticide-treated plant parts (Khan, 2020b; Sánchez-Bayo, Belzunces & Bonmatin, 2017). Consequently, in response to persistent or frequent exposure to insecticides, development and growth of target and nontarget species may be altered (Fernandes et al., 2016) or these species may develop resistance to insecticides due to selection pressure (Chouaïbou et al., 2016; Nkya et al., 2014). The development of insecticide resistance further worsens the problem of nontarget exposures and environmental pollution since farmers start over dosage of insecticides while ignoring the label recommendations in order to manage resistant insects (Khan, 2021a; Matowo et al., 2020).

Rice, *Oryza sativa* L., is the second most important staple food crop of Pakistan after wheat (*Triticum aestivum* L.), with an average 3,000 hectares cultivated annually (Abbas & Mayo, 2021). The management of the insect pests of rice crops is of utmost importance to ensure high yields; therefore, farmers use insecticides as one of the major insect pest management tools in Punjab, Pakistan (Asghar et al., 2013). The use of insecticides during the rice cropping season results in lethal and sublethal exposures to the environment and nontarget organisms on the premises (Chouaïbou et al., 2016). *Aedes albopictus* (Skuse) is one of the most common aquatic nontarget species present in the rice fields because the standing water requirement, particularly during the vegetative phase of the crop, provides an excellent habitat for its development (Preechaporn, Jaroensutasinee & Jaroensutasinee, 2007; Wan-Norafikah, Chen & Sofian-Azirun, 2021). *Aedes albopictus* is a holometabolous insect with four developmental stages including egg, larva, pupa and adult. Of these, the first three stages are aquatic while adults are aerial but females prefer to lay eggs in standing water or moist places (Anoopkumar et al., 2017). Most of the insecticides used in the rice crop in Punjab are either applied to the soil/water in granular form or in the form of foliar sprays (Table 1). In this way, different stages of *Ae. albopictus* might be exposed to insecticide residues through, drift, leaching, runoff and/or respiration in the contaminated air. Exposure of non-target species to insecticide residues in rice ecosystems increase the likelihood of resistance development to insecticides and negative effects on their biological traits (Fahad et al., 2015; Khan, 2020b).

**Table 1:** Insecticides tested against *Aedes albopictus* based on their use in rice farming system in Punjab, Pakistan (Ali, 2018).

Sr. #	Class	Insecticide	In use since	Target insect pest(s) in rice fields	Mode of action (IRAC)
1	Pyridine	Flonicamid 50WG	2017	Green leaf hopper, white backed plant hopper, & brown plant hopper	Chordotonal organ modulators -undefined target site
2	Diamide	Chlorantraniliprole 04G	2014	Leaf folder	Ryanodine receptor modulators
3	Pyrethroid	Gamma-cyhalothrin 60SC	2013	White backed plant hopper	Sodium channel modulators
4	Phenylpyrazole	Fipronil 03G	2011	White backed plant hoppers & stem borers	GABA-gated chloride channel blockers
5	Nereistoxin	Monomehypo 10G	2006	Stem borers	Nicotinic acetylcholine receptor channel blockers
6	Organophosphate	Triazophos 40EC	1999	Leaf folder & stem borers	Acetylcholinesterase inhibitors
7	Carbamate	Carbofuran 3G	1974	White backed plant hopper	Acetylcholinesterase inhibitors

Therefore, the present study was designed with the hypothesis that insecticidal usage in rice fields results in resistance to insecticides and exerts indirect effects on biological traits of nontarget *Ae. albopictus*.

## Materials and Methods

### Insects

**Figure 1:**
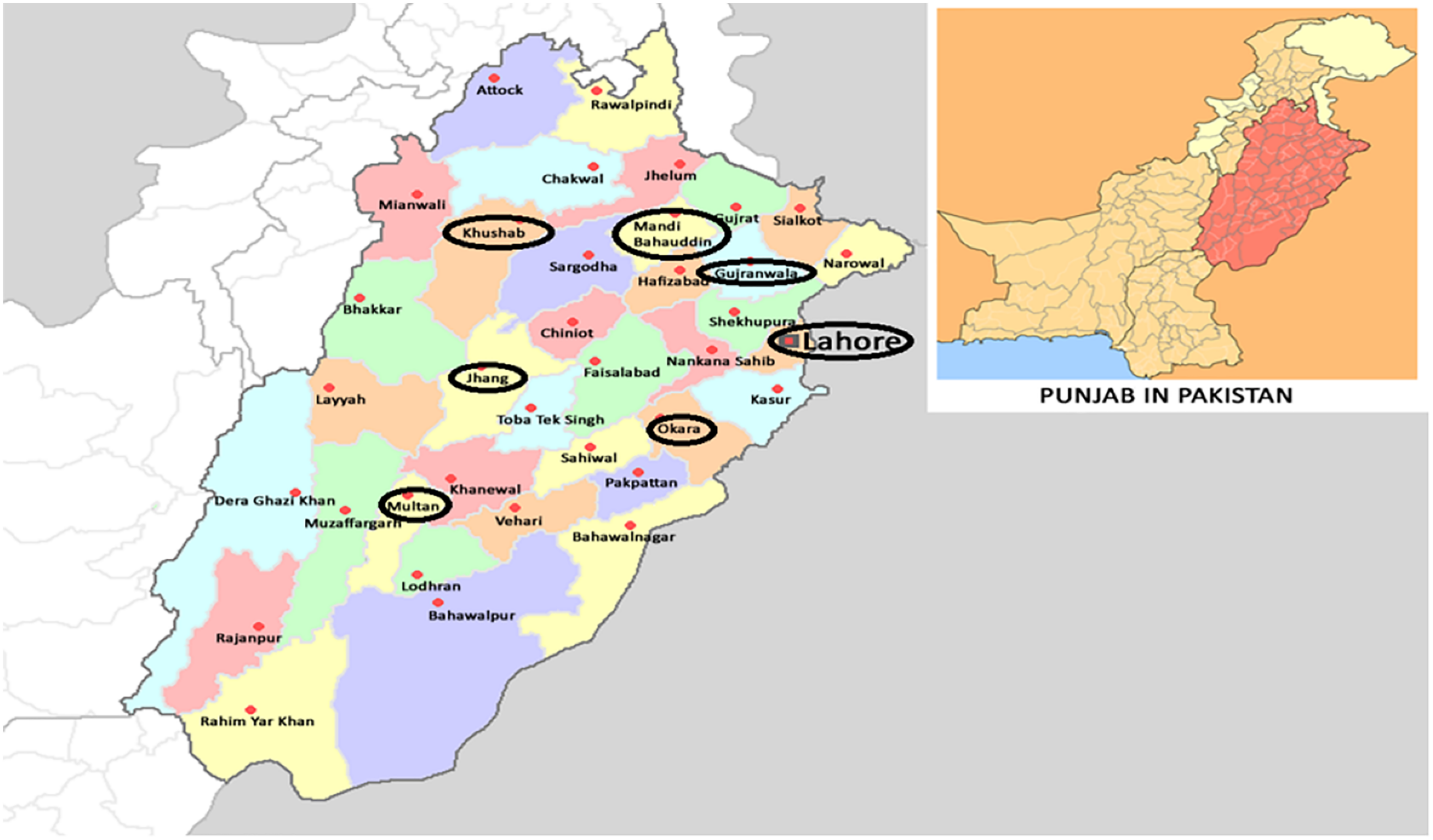
Collection areas of *Aedes albopictus* strains from major rice growing areas of Punjab, Pakistan (Wikimedia commons: https://commons.wikimedia.org/wiki/File:Pakistan_Punjab.png).

Field strains of *Ae. albopictus* from rice fields in major rice producing areas of Punjab, Pakistan (Fig. 1), were collected for experiments. Six field strains: OKR, GJR, MBD, KSB, MTN and JHG were collected from Okara (30.8138° N, 73.4534° E), Gujranwala (32.1877° N, 74.1945° E), Mandi Bahauddin (32.5742° N, 73.4828° E), Khushab (32.2955° N, 72.3489° E), Multan (30.1575° N, 71.5249° E) and Jhang (31.2781° N, 72.3317° E), respectively. The rice fields in these areas were selected based on a history of insecticides application with an average of four to five applications per cropping season (personal communication with farmers). A field strain (LHR) was collected from a rice field in Lahore (31.5204° N, 74.3587° E), where rice was produced with no, or minimal, usage of chemicals/insecticides. The REF strain was an insecticide-susceptible-strain cultured without exposure to insecticides (Khan, 2020a). All field strains were collected at the larval stage during 2018–2019 and cultured at 26 ± 1 °C, 65 ± 5% R.H. and 12:12 h photoperiod for at least three generations before starting bioassays. Field strains were collected from standing water in rice fields after nursery-transplantation stage. At least 500 larvae of each strain were kept separately in plastic basins containing one-liter distilled water. Larvae were fed with fish food (TetraMin®) at the rate of one-gram/plastic basin after every 2 days. The resulting pupae were transferred to wooden mesh cages (30 × 30 × 30 cm) for adult emergence. Adult *Ae. albopictus* were fed with a 10% (w/v) sugar solution. An anesthetized mouse (*Mus musculus* L.) was presented in each cage thrice a week as a source of blood-meal to female *Ae. albopictus*. In addition, a filter paper in a partially filled glass-beaker (250-ml) with water was introduced in each cage for egg deposition.

### Insecticide resistance detection bioassays

Commercial formulations of insecticides that are typically used in rice farming in Punjab, Pakistan (Ali, 2018; Khan, 2020b), were chosen for resistance detection in field strains of *Ae. albopictus* (Table 1). Concentration-response larval bioassays were performed following the guidelines of the World Health Organization (WHO, 2005) and an established protocol for resistance detection using mosquito larvae (Khan, 2020a; Khan & Akram, 2019; Khan et al., 2011). Briefly, late 3^rd^ or early 4^th^ instar larvae were exposed to a range of five to six concentrations (providing mortality between 2% to 98%) in three replicates, and 25 larvae per replicate. Control bioassays consisted of exposure of larvae to distilled water only. Each bioassay was performed three times in different days by preparing fresh insecticide solutions in distilled water. Mortality data were recorded after 24 h of exposure by observing the movement of the larvae after touching with a probe.

### Biological traits

Biological traits of all the strains of *Ae. albopictus*, such as the kinetics of larval development, survival rate of adults, rate of egg laying females (fertility) and fecundity, were studied separately following the methodology of Martins et al. (2012) with a few modifications. Briefly, 50 newly emerged 1^st^ instar larvae of each strain were taken in the plastic basin having 500 ml distilled water. The larvae were fed and reared as described above. The resulting pupae were transferred to wooden mesh cages (30 × 30 × 30 cm) for adult emergence. Upon adult emergence from pupae, the ratio of male and female mosquitoes was recorded. Adult mosquitoes were fed with 10% (w/v) sugar-water-solution, and the rate of survival of different stages was determined daily till the end of the cycle. All these bioassays were repeated six times.

Reproductive parameters such as the rate of egg laying females and the number of eggs per female (fecundity) of different strains were determined by taking newly emerged females (*n* = 20) from the above bioassays. These females were kept with males (1:1) in mesh cages for 5 days before receiving the first blood meal of the anesthetized mouse as described above. Three days after the blood meal, oviposition of engorged female mosquitoes was individually stimulated in glass petri-plates (6 cm diameter) covered with wet filter paper, and the number of egglaying females was recorded after 24 h (Martins et al., 2012; Valencia, Miller & Mazur, 1996) Number of eggs per female was recorded until the death of each female.

### Data analyses

Mortality data of each bioassay was subjected to Probit analysis using the PoloPlus software (LeOra-Software, 2005) to calculate the median lethal concentration (LC_50_) of each insecticide in each strain. By this analysis, median lethal concentrations (LC_50s_) of each strain against each insecticide were calculated. LC_50_ values of REF and LHR strains against all the insecticides were the lowest, suggesting that these strains were the most susceptible (Robertson et al., 2007) in comparison to the rest of field strains. Hence, LC_50_ values of these strains were used to calculate resistance ratios (RRs) of the rest of field strains against all tested insecticides by dividing LC_50_ of the field strain by that of REF or LHR strain. RR values were scaled using the following widely accepted criteria: “susceptibility (RR = 1), very low resistance (RR = 2–10), low resistance (RR = 11–20), moderate resistance (RR = 21–50), high resistance (RR = 51–100), and very high resistance (RR > 100)” (Ahmad, Arif & Ahmad, 2007; Khan, 2020b).

Data of biological traits (pupae formation, survival rate of male and female mosquitoes, rate of egglaying females, and fecundity) of all strains were analyzed by ANOVA, followed by Tukey’s multiple comparison test, using the Statistix v8.10 software (Statistix, 2005).

## Results

### Baseline toxicity of reference strains

Toxicity responses of REF and LHR strains of *Ae. albopictus* to different insecticides were used to assess variation in toxicity in the rest of the field strains (Table 2). Based on LC_50_ values, the REF strain was the most susceptible strain followed by the LHR strain. In the case of REF strain, the LC_50_ values different insecticides were: 0.39 mg/L for flonicamid, 0.61 mg/L for chlorantraniliprole, 0.21 mg/L for gamma-cyhalothrin, 0.54 mg/L for fipronil, 0.39 mg/L for monomehypo, 0.20 mg/L for triazophos, and 0.22 mg/L for carbofuran. The LC_50_ values of the LHR strain were: 7.65 mg/L for flonicamid, 2.86 mg/L for chlorantraniliprole, 3.19 mg/L for gamma-cyhalothrin, 8.88 mg/L for fipronil, 12.78 mg/L for monomehypo, 3.27 mg/L for triazophos, and 17.49 mg/L for carbofuran. In comparison to the REF strain, the LHR strain exhibited a very low level of resistance (RR = 4.69 fold) to chlorantraniliprole; low resistance to flonicamid (RR = 19.62 fold), gamma-cyhalothrin (RR = 15.19 fold), fipronil (RR = 16.44 fold) and triazophos (RR = 16.35 fold); moderate levels of resistance to monomehypo (RR = 32.77 fold) and high resistance to carbofuran (RR = 79.50 fold).

**Table 2:** Toxicity response of laboratory and field strains of *Aedes albopictus* to insecticides used in rice fields in Punjab, Pakistan.


**Insecticide**	**Strain**	n*	**LC**_**50**_ (95% CI)**	**Fit of probit line**				**RR** ^**£**^	**RR** ^**££**^
				**Slope (SE)**	***χ*** ^**2**^	**df**	***p***		
Flonicamid	REF	525	0.39 [0.34–0.45]	2.52 (0.35)	2.68	5	0.75	1	
	LHR	525	7.65 [6.63–8.85]	2.37 (0.41)	3.93	5	0.56	19.62 [16.04–24.16]	1
	OKR	525	22.59 [19.02–27.05]	1.80 (0.15)	1.84	5	0.87	57.92 [46.30–73.08]	2.95 [2.35–3.72]
	GJR	525	22.14 [18.07–27.44]	1.45 (0.13)	3.77	5	0.58	56.77 [44.22–73.49]	2.89 [2.24–3.74]
	MBD	525	26.52 [19.94–35.58]	1.90 (0.52)	5.70	5	0.34	68.00 [54.72–85.19]	3.47 [2.78–4.33]
	KSB	600	64.96 [48.89–89.23]	1.84 (0.14)	8.39	6	0.21	166.56 [133.41–2.09.69]	8.49 [6.77–10.67]
	MTN	600	58.09 [44.59–78.15]	1.53 (0.12)	5.67	6	0.46	148.95 [116.95–191.29]	7.59 [5.93–9.73]
	JHG	525	30.29 [21.11–44.89]	1.67 (0.35)	7.52	5	0.18	77.67 [61.61–98.71]	3.96 [3.13–5.02]
Chlorantraniliprole	REF	525	0.61 [0.42–0.83]	2.65 (0.23)	5.98	5	0.31	1	
	LHR	525	2.86 [2.44–3.35]	2.05 (0.16)	3.75	5	0.59	4.69 [3.53–5.89]	1
	OKR	525	13.76 [11.59–16.39]	1.81 (0.47)	3.42	5	0.64	22.56 [18.14–28.43]	4.81 [3.80–6.10]
	GJR	450	19.72 [16.44–24.70]	2.10 (0.22)	1.02	4	0.91	32.33 [25.45–41.67]	6.90 [5.33–8.92]
	MBD	525	58.59 [47.32–75.82]	1.55 (0.35)	3.18	5	0.67	96.05 [73.52–127.26]	20.49 [15.43–27.22]
	KSB	450	20.45 [17.09–24.76]	1.81 (0.47)	2.25	4	0.69	33.52 [26.75–42.62]	7.15 [5.60–9.13]
	MTN	600	42.80 [35.34–52.57]	1.50 (0.12)	2.73	6	0.84	70.16 [55.31–90.27]	14.97 [11.59–19.33]
	JHG	600	10.81 [9.07–12.72]	1.93 (0.15)	0.93	6	0.99	17.72 [14.30–22.27]	3.78 [2.99–4.77]
Gamma-cyhalothrin	REF	450	0.21 [0.12–0.33]	2.82 (0.29)	3.60	4	0.46	1	
	LHR	525	3.19 [2.20–4.74]	2.24 (0.17)	10.75	5	0.06	15.19 [12.20–18.51]	1
	OKR	525	15.89 [12.31–21.30]	2.09 (0.17)	5.25	5	0.39	75.67 [60.08–92.93]	4.98 [3.98–6.23]
	GJR	525	47.34 [39.67–56.30]	1.77 (0.40)	3.03	5	0.70	225.43 [177.55–278.94]	14.84 [11.77–18.71]
	MBD	525	29.10 [24.25–35.49]	1.69 (0.15)	1.33	5	0.93	138.57 [107.82–173.51]	9.12 [7.15–11.63]
	KSB	450	10.82 [9.19–12.70]	2.11 (0.39)	2.77	4	0.60	51.52 [41.01–63.12]	3.39 [2.72–4.24]
	MTN	450	18.28 [15.84–21.27]	2.49 (0.22)	1.18	4	0.88	87.05 [70.02–105.45]	5.73 [4.64–7.08]
	JHG	525	32.99 [25.63–44.02]	2.08 (0.17)	5.07	5	0.41	157.10 [124.63–193.02]	10.34 [8.26–12.95]
Fipronil	REF	525	0.54 [0.36–0.73]	2.32 (0.22)	4.63	5	0.46	1	
	LHR	525	8.88 [7.49–10.54]	1.83 (0.15)	2.17	5	0.83	16.44 [13.04–20.99]	1
	OKR	450	417.40 [334.82–564.29]	1.96 (0.42)	2.59	4	0.63	772.96 [573.78–1054.28]	47.00 [34.54–64.00]
	GJR	450	71.90 [60.51–86.02]	1.91 (0.19)	1.85	4	0.76	133.15 [105.28–170.48]	8.10 [6.33–10.36]
	MBD	525	101.58 [78.53–137.86]	2.26 (0.19)	5.64	5	0.34	188.11 [150.13–238.62]	11.44 [9.03–14.50]
	KSB	525	63.69 [46.93–89.38]	2.07 (0.36)	7.43	5	0.19	117.94 [94.20–149.49]	7.17 [5.66–9.09]
	MTN	525	39.40 [30.30–51.01]	1.80 (0.29)	4.36	5	0.50	72.96 [57.77–93.32]	4.44 [3.47–5.67]
Monomehypo	REF	450	0.39 [0.26–0.50]	2.33 (0.25)	3.09	4	0.54	1	
	LHR	525	12.78 [9.63–17.18]	1.75 (0.14)	4.88	5	0.43	32.77 [25.50–43.15]	1
	OKR	450	224.14 [157.72–362.82]	1.87 (0.29)	4.37	4	0.36	574.72 [442.91–764.70]	17.54 [13.41–22.95]
	GJR	450	111.27 [80.49–153.74]	2.82 (0.24)	6.04	4	0.20	285.31 [229.32–364.03]	8.71 [6.95–10.92]
	MBD	450	185.47 [134.78–269.42]	2.71 (0.45)	6.07	4	0.19	475.56 [380.53–609.48]	14.51 [11.53–18.28]
	KSB	525	119.52 [104.49–137.13]	2.62 (0.20)	3.63	5	0.60	306.46 [245.66–392.03]	9.35 [7.45–11.76]
	MTN	525	195.79 [168.12–231.54]	2.28 (0.20)	1.99	5	0.85	502.03 [396.58–651.69]	15.32 [12.02–19.55]
	JHG	525	127.56 [107.75–152.45]	1.85 (0.15)	2.40	5	0.79	327.08 [256.02–428.50]	9.98 [7.76–12.86]
Triazophos	REF	525	0.20 [0.17–0.24]	2.88 (0.17)	3.56	5	0.61	1	
	LHR	525	3.27 [2.79–3.85]	2.00 (0.36)	1.69	5	0.89	16.35 [12.70–20.12]	1
	OKR	525	77.16 [66.10–90.16]	2.10 (0.55)	2.43	5	0.77	385.80 [300.57–472.76)	23.60 [18.83–29.55]
	GJR	525	99.52 [86.28–115.39]	2.37 (0.19)	2.95	5	0.71	497.60 [390.28–605.60]	30.00 [24.45–37.85]
	MBD	450	51.13 [42.41–61.67]	1.75 (0.18)	2.11	4	0.72	255.65 [194.82–320.30]	15.63 [12.21–20.02]
	KSB	525	81.42 [54.56–125.63]	2.07 (0.39)	10.21	5	0.07	407.10 [316.67–499.56]	25.02 [19.84–31.22]
	MTN	525	44.04 [36.44–53.49]	2.78 (0.21)	4.18	5	0.52	220.20 [174.32–265.47]	13.47 [10.92–16.59]
	JHG	525	36.25 [26.40–50.30]	2.38 (0.18)	9.27	5	0.10	181.25 [142.37–220.30]	11.09 [8.92–13.77]
Carbofuran	REF	450	0.22 [0.18–0.26]	2.64 (0.25)	1.24	4	0.87	1	
	LHR	525	17.49 [13.09–23.40]	2.58 (0.19)	8.38	5	0.14	79.50 [64.13–99.21]	1
	OKR	525	113.56 [94.25–135.09]	1.79 (0.15)	1.15	5	0.95	516.18 [404.35–663.60]	6.49 [5.17–8.15]
	GJR	525	200.29 [168.67–238.58]	1.81 (0.14)	3.35	5	0.65	910.41 [716.42–1164.98]	11.45 [9.17–14.31]
	MBD	525	206.74 [159.07–271.44]	2.10 (0.46)	5.61	5	0.35	939.73 [748.62–1187.84]	11.82 [9.59–14.57]
	KSB	450	137.90 [115.69–165.29]	1.87 (0.18)	1.62	4	0.81	626.82 [491.86–804.39]	7.88 [6.29–9.88]
	MTN	525	133.37 [101.68–172.54]	2.02 (0.16)	5.16	5	0.40	606.23 [481.25–768.99]	7.63 [6.17–9.44]
	JHG	525	157.83 [136.81–181.98]	2.34 (0.18)	3.54	5	0.62	717.41 [576.52–898.96]	9.02 [7.40–11.02]

**Notes:**

An asterisk (*) indicates the number of insects used in bioassays.

Two asterisks (**) indicate the median lethal concentration in mg/L.

£ indicates the resistance ratio calculated by dividing LC50 of any strain by the LC50 of the REF strain.

££ indicates the resistance ratio calculated by dividing LC50 of any strain by the LC50 of the LHR strain.

### Resistance in field strains of *Ae. albopictus* in comparison to REF and LHR strains

The field strains of *Ae. albopictus* (OKR, GJR, MBD, KSB, MTN and JHG) exhibited significantly reduced toxicity to all tested insecticides compared with those of the REF and LHR strain (non-overlapped 95% CIs of LC_50s_) (Table 2). The LC_50s_ ranged from: 22.14–64.96 mg/L for flonicamid, 10.81–58.59 mg/L for chlorantraniliprole, 10.82–47.34 mg/L for gamma-cyhalothrin, 39.40–417.40 mg/L for fipronil, 111.27–224.14 mg/L for monomehypo, 36.25–99.52 mg/L for triazophos, and 113.56–200.29 mg/L for carbofuran. Due to shortage of suitable number of larvae, fipronil bioassays could not be performed against the JHG strain. Compared to the REF strain, these strains (OKR, GJR, MBD, KSB, MTN and JHG) revealed low to high levels of resistance to chlorantraniliprole; high to very high levels of resistance to flonicamid, gamma-cyhalothrin and fipronil; very high levels of resistance to monomehypo, triazophos and carbofuran. Whereas, these strains showed very low levels of resistance to flonicamid; very low to low resistance to chlorantraniliprole, gamma-cyhalothrin, monomehypo and carbofuran; very low to moderate resistance to fipronil; low to moderate resistance to triazophos, compared with the LHR strain of *Ae. albopictus*.

### Biological traits of reference and field strains of *Ae. albopictus*

**Figure 2:**
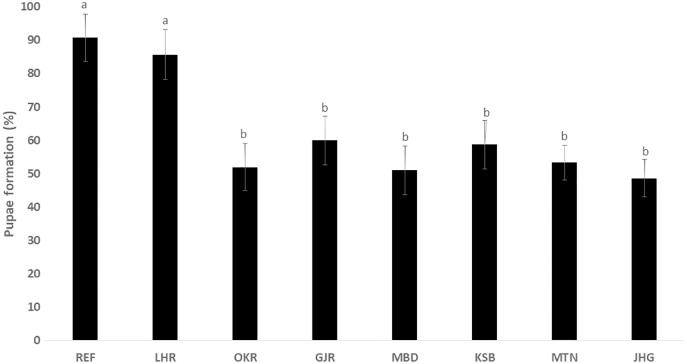
Percentage of larvae of different strains of *Aedes albopictus* converted to pupal stage (mean ± S.E.).

**Figure 3:**
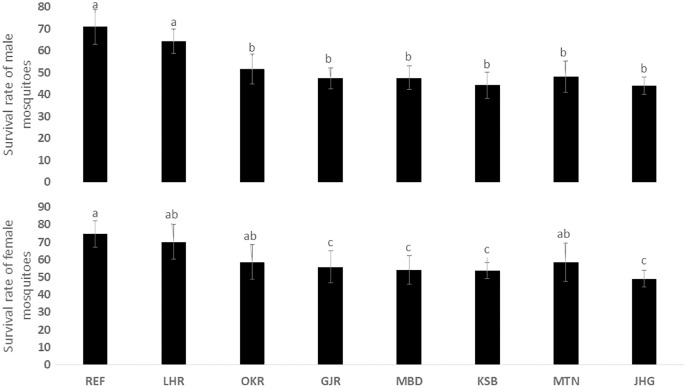
Survival rate (%) of male and female adults of different strains of *Aedes albopictus* (mean ± S.E.).

**Figure 4:**
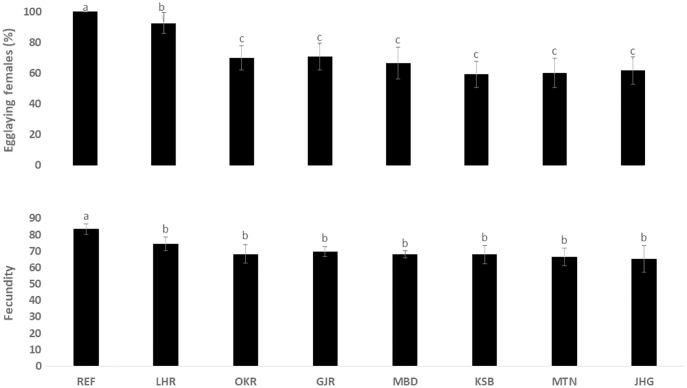
Rate of blood females (%) that deposited eggs, and fecundity (number of eggs per female) of different strains of *Aedes albopictus* (mean ± S.E).

The rate of pupal formation (%) was reduced significantly in the field strains of *Ae. albopictus* (OKR, GJR, MBD, KSB, MTN and JHG) when compared with REF and LHR strains (F = 34.10; df = 7, 40; *p* < 0.01; Fig. 2). The rate of pupae formation in the REF and LHR strains was 90.67 and 85.67%, respectively, while the rest of the field strains exhibited 51–60% pupae formation rate. There was an obvious decline in the survival rate (%) of male and female (except females of OKR and MTN strains) mosquitoes of field strains compared to the male and female mosquitoes of REF and LHR strains (F = 15.70; df = 7, 40; *p* < 0.01 for the male survival rate; F = 6.40; df = 7, 40; *p* < 0.01 for the female survival rate; Fig. 3). Similarly, significant reduction in the number of egglaying females (%) was observed in field strains compared with those of the reference strains; however, fecundity of all field strains was only significantly different with that of the REF strain (F = 21.40; df = 7, 40; *p* < 0.01 for egglaying females; F = 8.40; df = 7, 40; *p* < 0.01 for fecundity; Fig. 4).

## Discussion

There has long been a concern about the use of insecticides in crop farming systems and their influence on nontarget organisms such as development of resistance to insecticides and impaired biological traits (Antonio-Nkondjio et al., 2008; Nkya et al., 2014). The standing water requirement in the early vegetative phase of the rice crop provides an excellent environment for mosquitoes’ breeding (Djègbè et al., 2020). Mosquito larvae in the standing water are hypothesized to get continuous exposure to insecticides used on the rice crop mainly due to leaching or contamination of breeding sites and become resistant to insecticides over time (Reid & McKenzie, 2016). Moreover, insecticidal usage in crop farming may alter the performance of biological parameters of nontarget species present in the premises, which get direct or indirect exposure to insecticide residues largely due to their life activities such as feeding, breeding and/or seeking shelter in cropping areas (Khan, 2020b; Khan et al., 2011). The present study was aimed to check these hypotheses in six field strains of *Ae. albopictus* absolutely collected from the rice farming system with heavy usage of insecticides and compared their resistance and biological traits with two reference strains of *Ae. albopictus*. Overall, the results revealed significant levels of resistance to insecticides and the weaker performance of biological traits compared with those of the reference strains. These results clearly demonstrate the possible side effects of insecticides used in rice farming on the nontarget field strains of *Ae. albopictus*.

The findings of current study confirmed a field evolved resistance in the collected *Ae. albopictus* strains from Punjab rice fields. Previously, there are different reports of resistance development in different mosquito species (*e.g*., *An. gambiae*, *Ae. albopictus*, *An. stephensi*) as a result of the use of insecticides in cropping areas (Diabate et al., 2002; Hien et al., 2017; Khan, Akram & Lee, 2018; Khan et al., 2011; Nkya et al., 2014). Insecticidal bioassays confirmed that field strains of *Ae. albopictus* showed varying levels of field-evolved resistance to insecticides used in rice farming systems. REF and LHR strains showed the highest susceptibility to all tested insecticides, most probably due to no or very low chemical exposure, when compared with the rest of the strains. Compared to the REF strain, these strains (OKR, GJR, MBD, KSB, MTN and JHG) revealed low to high levels of resistance to chlorantraniliprole; high to very high levels of resistance to flonicamid, gamma-cyhalothrin and fipronil; very high levels of resistance to monomehypo, triazophos and carbofuran. Whereas, these strains showed very low levels of resistance to flonicamid; very low to low resistance to chlorantraniliprole, gamma-cyhalothrin, monomehypo and carbofuran; very low to moderate resistance to fipronil; low to moderate resistance to triazophos, when compared with the LHR strain of *Ae. albopictus*. All these insecticides are intensively used for the management of insect pests in rice farming systems (Khan, 2020b). Field evolved resistance to insecticides is an example of contemporary evolution where species show adaptive evolutionary changes in responses to external stimuli with the passage of time (Stockwell, Hendry & Kinnison, 2003).

The use of insecticides in cropping areas often have lethal and sublethal effects on target and nontarget species, which may lead to the development of insecticide resistance (Guedes, Walse & Throne, 2017). Recently, varying levels of resistance to insecticides used in rice farming have been reported in the nontarget house fly, *Musca domestica* L., collected from rice farms of Punjab, Pakistan (Khan, 2020b). For instance, field strains of *M. domestica*, in comparison to a laboratory susceptible strain, exhibited 7.83 to 13.28 fold resistance to flonicamid, 11.13 to 19.83 fold resistance to triazophos, 19 to 43 fold resistance to gamma-cyhalothrin, 13.23 to 40.15 fold resistance to fipronil, 11.90 to 27.10 fold resistance to chlorantraniliprole and 14.38 to 25.84 fold resistance to monomehypo. Previously, most of the tested insecticides in present study caused toxicity to nontarget species. For example, fipronil has been reported hazardous to nontarget insect species when used to control grasshoppers (Balança & De Visscher, 1997). Carbofuran significantly reduced populations of nontarget dragonflies, damselflies, coccinellid beetles and carabid beetles when used to control insect pests in the rice crop (Srinivas & Madhumathi, 2005). Flonicamid showed negative effects on the nontarget natural enemies (*Amblyseius swirskii* Athias-Henriot and *Nesidiocoris tenuis* Reuter) of *Bemisia tabaci* (Gennadius) (Roditakis et al., 2014). Similarly, chlorantraniliprole showed acute toxicity to the nontarget crayfish *Procambarus clarkii* Girard associated with rice–crayfish cropping systems (Barbee et al., 2010).

The use of insecticides in the cropping ecosystem usually result in lethal and sublethal exposures to nontarget species in the premises that generally cause either direct mortality or affect the performance of biological traits (Desneux, Decourtye & Delpuech, 2007; Khan, 2021b). Results of the present study revealed that field strains of *Ae. albopictus* exhibited significantly reduced rates of pupal formation, survival of male and female mosquitoes, rate of egglaying females and fecundity, in comparison to LHR and REF strains. Since field strains showed resistance to all tested insecticides, the weak performance of the biological traits may be due to the cumulative effect of exposure to different insecticides in the field. Sublethal exposures to insecticides in the field cause certain physiological or biochemical changes in exposed organisms, which ultimately affect the performance of biological traits (Fernandes et al., 2016). Additionally, the production of detoxifying enzymes (*e.g*., transferases, esterases) is a general phenomenon by exposed organisms to dilute the negative effects of insecticides, which usually require a lot of energy to produce such enzymes. Resultantly, energy required for development and growth suffer deficiency that lead to the weak performance of biological traits (David et al., 2018).

Nontarget species in the premises of cropping areas get direct or indirect exposures to insecticide residues during their routine life activities such as seeking food, shelter and space (Khan, 2021c). Most of the tested insecticides in the present study are systemic (except triazophos and gamma-cyhalothrin) and contact poisons that are applied in the form of granules (a dry formulation) in the rice crop. Being systemic in nature, these insecticides readily translocate in soil and plant parts such as roots, stems, leaves, flowers, nectar, pollens and guttation drops (Stoner & Eitzer, 2012). Triazophos and gamma-cyhalothrin are used as liquid spray applications on the rice crop in Punjab, Pakistan (Khan, 2020b). Residues of these insecticides also contaminate different plant parts and surrounding air right after spray applications. Hence, the nontarget *Ae. albopictus* strains have high probability to get insecticide exposure through: flight activities of adults in the cropping area, feeding of adults on flower nectar, oviposition in the standing water in the rice crop, and/or during aquatic-immature stages (egg, larva, pupa) in the rice crop.

## Conclusions

In view of the results presented here, it can be concluded that the use of insecticides in rice crops significantly affected the nontarget *Ae. albopictus* that is reflected in the form of resistance development to insecticides and altered performance of selected biological traits. Since *Ae. albopictus* is an important vector of dengue fever in many parts of the world, including Pakistan, it would be very difficult for pest managers to manage resistant *Ae. albopictus* with mosquitocides if there is a cross-resistance phenomenon.

## Supplemental Information

10.7717/peerj.13697/supp-1 Supplemental Information 1 Raw data. Insecticidal bioassays data and life history parameters.

## Additional Information and Declarations

### Data Availability

The following information was supplied regarding data availability: The raw measurements are available in the Supplemental File.
